# Chemically synthesized high-fidelity oligos ≤ 600 nt as building blocks to accelerate complex gene construction in synthetic biology

**DOI:** 10.1093/synbio/ysag005

**Published:** 2026-05-23

**Authors:** Mancang Zhang, Yang Hu, Hao Huang, Yongyong Shi

**Affiliations:** Bio-X Institutes, Key Laboratory for the Genetics of Developmental and Neuropsychiatric Disorders (Ministry of Education), Shanghai Jiao Tong University, Shanghai 200030, People's Republic of China; Shanghai Dynegene Technologies Co., Ltd., R&D Department, Shanghai 201108, People's Republic of China; United Research Center for Next Generation DNA Synthesis of SJTU, Innovation Team, Shanghai 201108, People's Republic of China; United Research Center for Next Generation DNA Synthesis of SJTU, Innovation Team, Shanghai 201108, People's Republic of China; Bio-X Institutes, Key Laboratory for the Genetics of Developmental and Neuropsychiatric Disorders (Ministry of Education), Shanghai Jiao Tong University, Shanghai 200030, People's Republic of China; Institute of Neuroscience, Center for Excellence in Brain Science and Intelligence Technology, Chinese Academy of Sciences, Shanghai 200031, People's Republic of China

**Keywords:** ultralong oligonucleotides, chemical synthesis, complex genes, synthetic biological DNA parts

## Abstract

Synthetic biology and advanced genetic engineering applications rely heavily on the efficient construction of large and complex DNA sequences. Current DNA synthesis technologies have limited capacity to efficiently generate ultralong oligonucleotides for complex gene construction, particularly those with extensive repetitive motifs and uneven base distribution. Here, we report a novel platform named UCOS (Ultralong Complex Oligonucleotides Synthesis) that enables the efficient synthesis of long, complex, and challenging DNA fragments. This platform employs nonporous silica microspheres as the solid support instead of the traditional controlled pore glass solid support, full-length enrichment based on 5′ flank sequence hybridization and an error-removing enzyme for correct sequence selection, substantially enhancing the fidelity of intricate, ultralong oligonucleotides. Using this approach, we successfully synthesized challenging sequences ≤600 nt in length, encompassing tandem repeats and uneven base distributions. Overall, this novel platform demonstrates exceptional efficiency and reliability in handling ultralong DNA fragments with highly repetitive and complex features. This novel platform provides a strong foundation for advancing synthetic biology and metabolic engineering, showing great potential as a powerful tool for constructing challenging genes and enabling the customized synthesis of functional genetic elements for complex genetic programmes and synthetic genomics.

## Graphical Abstract

**Graphical Abstract:**
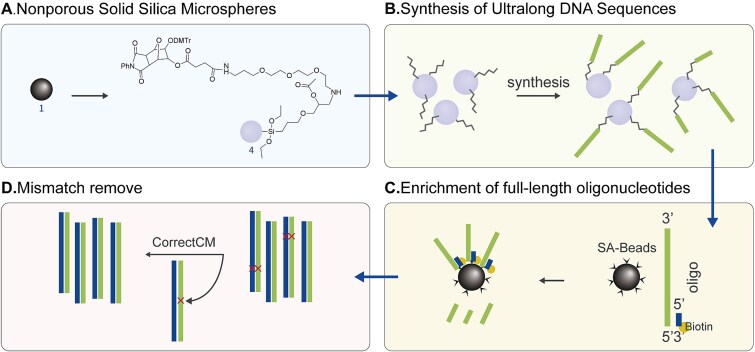

## Introduction

DNA, the fundamental molecule of life and carrier of genetic information, has been at the forefront of biotechnological advancement. The development of DNA synthesis technologies has revolutionized various fields, including synthetic biology [1], drug development [2], and gene therapy [3]. Since the inception of oligonucleotide chemical synthesis research in the 1950s [4], significant progress has been made. The phosphodiester method was proposed by H. G. Khorana and was a common method for the early synthesis of oligonucleotides [5]. A notable breakthrough came in the 1980s when Marvin Caruthers reported the phosphoramidite method for oligonucleotide synthesis [6]. Currently, the solid-phase phosphoramidite method, developed by Beaucage and Caruthers, remains the most widely used approach, comprising four essential steps: deprotection, coupling, capping, and oxidation [7].

The evolution of oligonucleotide synthesis capabilities has opened new frontiers in molecular biology [8, 9]. The ability to synthesize 20–30 base sequences enabled the development of Polymerase chain reaction (PCR) and DNA sequencing, establishing foundations for recombinant DNA technology and molecular diagnostics. Furthermore, the capacity to synthesize 50–100 base sequences facilitated precise DNA manipulation techniques, including site-directed mutagenesis and genetic engineering.

However, chemical DNA synthesis has reached a plateau due to inherent limitations [10]. Current synthesis processes face challenges such as depurination [11] caused by chemical reagents and incomplete capping, making the synthesis of oligonucleotides > 200 nt in length particularly challenging [8, 12]. Longer DNA sequences are typically assembled through the ‘splicing’ of shorter oligonucleotides. The term ‘splicing’ used here refers to *in vitro* DNA fragment assembly (not RNA splicing), specifically the ligation or polymerase-mediated joining of ultralong synthesized oligos into full-length genes or genetic pathways. This process involves synthesizing fragments of <100 nt (typically 80 nt) with homologous ends, followed by assembly using techniques such as Polymerase Chain Assembly (PCA) [13, 14] or Gibson assembly [15–17]. The chemical synthesis of noncomplex green fluorescent protein (GFP) genes and DNA polymerase genes using a long-oligo-based strategy was reported in a recent work [18], but this catching-by-polymerization (CBP) method requires specialized chemical reagents and intricate gel purification, which limits its widespread adoption. Furthermore, this approach encounters difficulties with challenging sequences, particularly those containing long repetitive elements or unbalanced base composition.

Building upon these challenges in DNA synthesis, particularly for sequences with unique structural features, there is an urgent need for innovative solutions. The complexity of target sequences in modern biotechnology applications presents diverse synthesis challenges that require novel approaches.

Among the most demanding targets are sequences with extreme base composition bias and repetitive elements. For example, the *Plasmodium falciparum* genome, with its unusually high AT content (~80%–85%) [19], represents a fundamental challenge in synthesis fidelity and assembly. Similarly, spider silk protein genes, characterized by their extensive repetitive modules and substantial length (often >10 kb), contain multiple iterations of polyalanine blocks and glycine-rich regions that are crucial for biomaterial engineering applications [20]. The synthesis of tandem repeat (TR) sequences [21], essential for genetic fingerprinting and disease research, presents additional complexity due to their highly repetitive nature. Further challenges include G-quadruplex-forming sequences in telomeres [22], large structural RNAs [23], and synthetic genes containing complex secondary structures [24].

**Figure 1:**
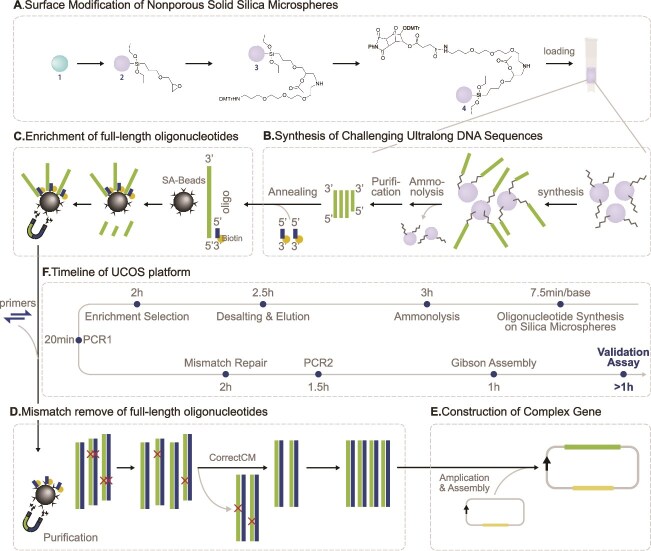
The workflow of the UCOS platform. (A). Schematic illustration of the surface modification of nonporous solid silica microspheres. Primary surface grafting of pretreated raw nonporous solid silica microspheres to yield intermediate (2); secondary grafting and capping of residual hydroxyl groups to prevent unwanted downstream reactions to produce intermediate (3); deprotection of DMT groups followed by condensation reaction with linker molecules to generate product (4); column packing with product 4 to prepare the solid support for subsequent oligonucleotide synthesis. (B). Schematic representation of ultralong DNA fragment synthesis occurring in the packed synthesis column. This synthesis process relies on the standard phosphoramidite cycle (including deprotection, coupling, capping, and oxidation steps) for nucleotide chain extension. After synthesis, the long DNA fragments are subjected to ammonolysis to cleave from the solid support, followed by desalting for purification. (C). Selection of full-length oligonucleotides. The purified fragments from step (B) are annealed with biotinylated primers (complementary to the 5′ termini of target sequences). Following annealing, full-length sequences are captured using streptavidin-coated magnetic beads; the captured full-length oligonucleotides are then amplified by PCR. (D) Mismatch removal of the enriched full-length oligonucleotides. The PCR-amplified fragments from step (C) are first annealed, and mismatch repair enzymes are subsequently used to eliminate error-containing sequences. (E). Schematic representation of vector construction. Following PCR amplification, the sequences were cloned into plasmid vectors for validation studies. (F) Schematic timeline of the entire UCOS platform workflow. This panel illustrates the chronological sequence of key steps in the UCOS platform, from nonporous silica microsphere functionalization and ultralong DNA synthesis to postsynthesis processing, full-length fragment capture, mismatch removal, and final Gibson assembly (total duration ~ 31 hours for a 600-nt complex sequence). Notably, PCR1 specifically refers to the low-cycle PCR amplification of full-length fragments enriched via streptavidin magnetic beads, which provides sufficient templates for subsequent mismatch removal; PCR2 denotes the high-fidelity PCR amplification of error-corrected fragments, ensuring the resulting fragment concentration meets the requirement for downstream vector assembly.

To address these specific challenges, we have developed a novel synthesis platform UCOS optimized for ultralong complex oligonucleotide synthesis. A schematic representation of the synthesis process is shown in Fig. 1. Innovatively, our approach uses nonporous solid silica microspheres as synthesis carriers (Fig. 1A and B), instead of controlled pore glass (CPG), which significantly enhances the coupling efficiency and stability of ultralong oligonucleotides. Furthermore, we use biotinylated primers to enrich full-length fragments and filter out incomplete products (Fig. 1C). Additionally, we employ mismatch repair enzymes to remove fragments containing synthesis errors, such as insertions, deletions, and base substitutions (Fig. 1D). The high-accuracy full-length oligonucleotides obtained are then further assembled into vectors for subsequent functional validation (Fig. 1E).

Using this platform, we have successfully synthesized challenging sequences ≤600 nt in length, including those with repetitive elements and unbalanced base composition. The biological functionality and sequence accuracy of these synthesized oligonucleotides have been thoroughly validated. This advancement represents a significant step forward in enabling applications across fundamental life science research, cellular and gene therapy, protein engineering, and, critically, synthetic genomics, genome engineering, and the rapid prototyping of genetic systems.

## Materials and methods

### Ultralong oligonucleotide synthesis

Our novel synthesis platform is based on nonporous solid silica microspheres for long-sequence DNA synthesis. The optimized preparation protocol for nonporous solid silica microspheres began with the pretreatment of these microspheres using 2 M ammonium fluoride solution to generate hydroxyl-rich surfaces. Subsequently, a primary grafting step was performed using 10% (w/w) glycidoxypropyltrimethoxysilane (GOPS) silane reagent in acetonitrile. The modified microspheres then underwent a secondary grafting process with DMT-diamine in the presence of alkylamine N,N-dimethylformamide (DMF), followed by deprotection using trichloroacetic acid to remove protecting groups. The final functionalization step involved a condensation reaction between the modified microspheres and linker molecules, Unylinker, in the presence of condensing agents (dimethyl sulfoxide (DMSO), N,N-diisopropylethylamine (DIPEA), and O-(benzotriazol-1-yl)-N,N,N′,N′-tetramethyluronium hexafluorophosphate (HBTU)). Notably, to prevent unwanted reactions between hydroxyl groups located at the intersection of primary and secondary grafting reagents and downstream linker molecules, we performed capping of these groups using acetic anhydride. This comprehensive surface modification protocol yielded nonporous solid silica microsphere supports suitable for oligonucleotide synthesis. The nonporous silica microspheres (solid nonporous structure, specific surface area 0.03–0.05 m^2^/g, average particle size 50–100 μm; protected by Chinese Patent CN 117229330B) were characterized via dynamic light scattering for size uniformity (polydispersity index < 0.1) and scanning electron microscopy (SEM) for nonporous morphology. Quantitative comparison with traditional CPG supports (500 Å pore size, 100–200 mesh) showed (i) oligonucleotide loading capacity: 0.5 nmol/cm^2^ (silica) versus 0.2 nmol/cm^2^ (CPG); (ii) synthesis reproducibility: coefficient of variation (CV) 4.2% (silica) versus 9.5% (CPG) for 400-nt poly(T) sequences; (iii) coupling efficiency at 600 nt: 99.5% (silica) versus 98.8% (CPG). These parameters confirm the microspheres’ superiority for ultralong complex DNA synthesis. Then we employed the standard phosphoramidite methodology for the synthesis of ultralong oligonucleotides on the DYHOW2B synthesis equipment (Dynegene Technologies) with our nonporous solid silica microspheres support columns. All the subsequent sequences were synthesized using our UCOS platform. For the 352-nt tandem repeat sequence, we also assessed commercial synthesis feasibility by submitting identical orders to two service providers: GenScript Biotech (vendor A, Nanjing, China) and Tsingke Biotechnology (vendor B, Beijing, China).

### Validation of ultralong oligonucleotides

We designed synthetic sequences based on genomic regions encompassing 200 base pairs upstream and downstream of selected expression short tandem repeat (eSTR) loci. The final synthetic sequences were constructed by inserting eSTRs [0, 17, 33, and 50 repeat units (rpt)] into the central region of the basic sequence. Synthesis was conducted in silica microsphere–packed columns, prepared according to the methodology described in the protocol for ultralong oligonucleotide synthesis. Synthesized sequences underwent desalting purification. The purity and fragment size of the synthesis products were verified using polyacrylamide gel electrophoresis (PAGE). An 8% polyacrylamide gel containing 7 M urea was prepared in 1× Tris-Borate-EDTA (TBE) buffer buffer. The gel solution was filtered through a 0.22 μm membrane and degassed for 15 minutes. Polymerization was initiated by adding 0.1% (w/v) ammonium persulfate (APS) and 0.01% (v/v) *N*,*N*,*N*′,*N*′-tetramethylethylenediamine. DNA samples (2–4 μl, 100 ng/μl) were mixed with an equal volume of 2× loading buffer (#AM8546G, Invitrogen, USA) and denatured at 100°C for 5 minutes, followed by immediate cooling on ice. A 50-bp DNA ladder (#ALH313, Balb, China) was used as a size marker. Electrophoresis was performed at 200 V constant power for 40 minutes in 1× TBE buffer at room temperature. The gel was stained with SYBR Gold (1:10 000 dilution in 1× TBE; #S11494, Invitrogen, USA) for 10–15 minutes in the dark with gentle agitation. After three washes with deionized water, the gel was visualized using a BluSight Pro system (#GD50502, Monad, China). Detailed information for the two eSTR loci synthetic sequences is provided in Table S1.

### Enrichment of full-length oligonucleotides

A complementary primer was designed to target the 5′ end of each synthetic sequence, with biotin conjugated to the primer’s 3′ terminus [25]. Primers and synthetic fragments were diluted in IDT Duplex buffer at equimolar ratios and subjected to the following annealing protocol: heating to 98°C and then gradually cooling to 60°C at a rate of 1°C per minute; incubating at 64°C for 10 min and then gradually cooling to room temperature at a rate of 1°C per minute. Biotinylated primers and their corresponding synthetic fragments were purified using QuarAcces Hyper Enrichment Beads (#ND3018, Dynegene, China).

**Figure 2:**
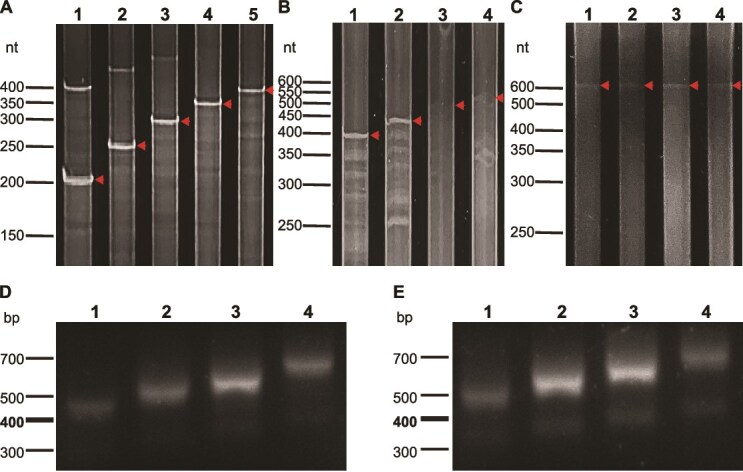
Validation of synthesized ultralong oligonucleotides. (A) PAGE analysis of multiple poly(T) samples, each containing a single fragment of different length (200–400-nt range). Lane 1: 200-nt poly(T) oligo. Lane 2: 250-nt poly(T) oligo. Lane 3: 300-nt poly(T) oligo. Lane 4: 350-nt poly(T) oligo. Lane 5: 400-nt poly(T) oligo. (B) PAGE analysis of synthesized eSTR-containing fragments derived from the *FRA10AC1* gene locus. Each lane represents a distinct fragment with varying lengths ranging from 400 to 550 nt. Lane 1: 400-nt oligo. Lane 2: 451-nt oligo with 17 repeats of eSTR locus. Lane 3: 499-nt oligo with 33 repeats of eSTR locus. Lane 4: 550-nt oligo with 50 repeats of eSTR locus. (C) PAGE analysis of synthesized 600-nt fragments containing 270 CT repeats. Each lane represents the product synthesized by an individual synthesis column (*n* = 4 technical replicates, confirming the stability of the UCOS platform). (D) Agarose gel electrophoresis analysis of biotinylated primer–captured DNA fragments. Lanes 1–4 correspond to the same synthetic oligonucleotide samples as in (B); the captured products were amplified to yield fragments of 400, 451, 499, and 550 nt, respectively. (E) Agarose gel electrophoresis analysis of mismatch repaired DNA fragments. Lanes 1–4 correspond to the same captured products as in (D). Following mismatch repair, amplification was performed using primers harbouring homology arms complementary to the minP-EGFP vector, resulting in extended fragment lengths of 446, 497, 545, and 596 nt, respectively.

**Figure 3:**
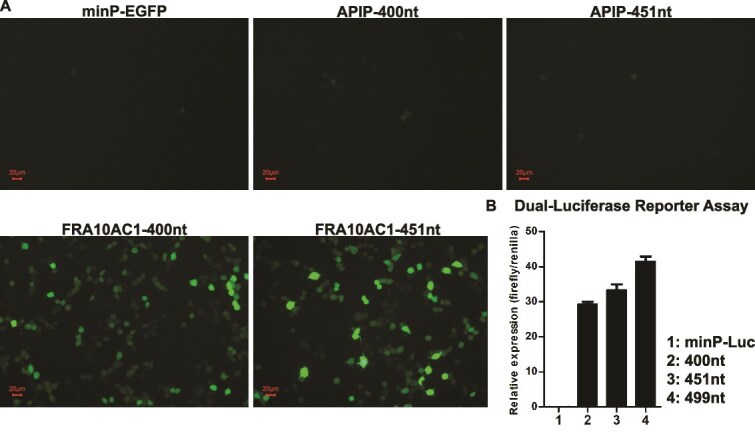
Functional validation of synthesized eSTR locus sequences. (A) Fluorescence microscopy imaging shows the effects of different eSTR loci on EGFP expression. *APIP*-associated loci showed no significant upregulation of downstream EGFP compared to the minP. *FRA10AC1*-associated loci demonstrated substantial upregulation of EGFP expression. (B) Dual-luciferase reporter assays reveal a strong correlation between the repeat number of *FRA10AC1*-associated eSTR loci and downstream luciferase expression. Luciferase expression levels increased progressively with the increasing number of eSTR repeats. Error bars represent SD, derived from two technical replicates in a single experimental run.

To confirm the efficiency and selectivity of this biotin-based enrichment procedure, we validated the recovery of full-length products using two orthogonal approaches: the biotin-enrichment procedure achieved selective recovery of full-length products, with the enrichment efficiency validated by PAGE (Fig. 2) and agarose gel electrophoresis (Fig. 3A). Specifically, PAGE analysis of pre-enrichment samples showed visible non-full-length bands (e.g. lower-molecular-weight fragments in Fig. 2B), while postenrichment agarose gel electrophoresis (Fig. 3A) revealed the reduced intensity of non-full-length bands and prominent target bands (400–550 nt), confirming that the procedure effectively enriches full-length products by removing incomplete synthesis by-products.

### Mismatch removal of full-length oligonucleotides

The purified fragments were amplified by PCR using the 2xPCR Mix (#NL4001, Dynegene, China) with specific primers for each eSTR. PCR amplification was performed under the following conditions: initial denaturation at 95°C for 30 s, followed by 10 cycles of denaturation at 95°C for 15 s, annealing at 60°C for 15 s, and extension at 72°C for 1 min. A final extension step was conducted at 72°C for 3 minutes. Then, the purified amplification products were prepared for error correction by adding 1 μl of 10× Reaction Buffer I (#NE1003, Dynegene, China) and nuclease-free water to a total volume of 10 μl, with initial PCR thermal cycling performed at 98°C for 2 minutes, followed by sequential steps at 4°C for 5 minutes, 37°C for 5 minutes, and a final hold at 4°C. After reaching the final hold step, 2 μl of Exonuclease X (#NE1003, Dynegene, China) and 6 μl of CorrectCM I (#NE1003, Dynegene, China) were added to the annealed product. The mixture was then incubated at 37°C for 1 hour, with the thermal cycler lid temperature set to 47°C. Following the previous reaction, a 1 μl aliquot of 100-fold-diluted CorrectCM II (#NE1003, Dynegene, China) was added. The sample was then incubated at 37°C for 30 minutes, with the thermal cycler lid temperature maintained at 47°C.

### Complex gene construction

The error-corrected product was then prepared for PCR amplification by adding fragment-specific primers (10 μM, 2 μL each), 25 μL of 2xPCR Mix (#NL4001, Dynegene, China), and nuclease-free water to a total volume of 50 μL. PCR amplification was conducted using the following thermal cycling conditions: an initial denaturation at 95°C for 3 minutes, followed by 25 cycles of denaturation at 95°C for 15 s, annealing at 60°C for 15 s, and extension at 72°C for 1 minute, with a final extension step at 72°C for 5 min and a terminal hold at 4°C. The amplified error-corrected product was purified with QuarAcces Hyper Pure Beads (#ND3011, Dynegene, China) and eluted with nuclease-free water and quantified using a Thermo NanoDrop OneC. It is worth noting that for sequences with high GC content, 5% DMSO was supplemented to the PCR reaction mixture. Detailed primer sequence information is provided in Table S2. All the primers were synthesized by Dynegene Technologies.

For the 5×HRE sequence, Gibson Assembly (#NG1002, Dynegene, China) was employed to insert the error-corrected fragments into the linearized vector carrying a complete downstream expression cassette. Sanger sequencing was used to assess the complete accuracy of the final sequence.

For the 510-nt GFP-derived sequence, Next-Generation Sequencing (NGS) library preparation was performed using the NGS Library Prep Kit (#ND620-02, Vazyme, China) followed by paired-end sequencing (2 × 250 bp). Concurrently, the synthetic 510-nt fragment was cloned into the pUC57 vector containing the necessary expression elements, which was then transformed into TOP10 competent cells (#DL1010M, Weidi, China). GFP expression was observed the next day for functional validation.

### Functional verification of complex gene construction based on ultralong oligonucleotide synthesis

We constructed a series of plasmids based on the pGL4 vector backbone, which were synthesized by Dynegene Technologies according to the sequence information available on the Addgene homepage. The plasmid minP-EGFP was generated by inserting a minimal promoter (minP) sequence and the enhanced green fluorescent protein (EGFP) reporter gene, with two SfiI restriction sites featuring different sticky ends inserted upstream of the minP. The linearized minP-EGFP plasmid was prepared using SfiI restriction enzyme (#R0123S, New England Biolabs, USA). Gibson Assembly (#NG1002, Dynegene, China) was employed to insert the error-corrected eSTR fragments into the linearized vector, using primers with homology arms corresponding to the vector's SfiI flanking sequences. Plasmids were extracted using an endotoxin-free plasmid extraction kit (#12163, QIAGEN, Germany) and transfected into HEK293T cells using polyethyleneimine (PEI; #24765-1, Polysciences, USA). EGFP expression was visualized via fluorescence microscopy 24 hours posttransfection.

We replaced the EGFP reporter gene with luciferase in the previously constructed plasmids. A *Renilla* Luciferase-expressing plasmid (#E6911, Promega, USA) was used as an internal control. HEK293T cells were cotransfected with luciferase and *Renilla* plasmids at a 50:1 ratio. Dual-luciferase activity was measured using a luminometer 24 hours posttransfection, following the detailed protocol provided in the Promega Dual-Luciferase Reporter Assay kit (#E1910, Promega, USA). The assay was performed in a single experimental run with two technical replicates; data were presented with error bars representing standard deviation (SD), normalized to *Renilla* luciferase activity to account for transfection efficiency variations.

### Material availability

All the materials and reagents used in this study were obtained from commercial sources, and the vendors are specified in the Materials and methods section of the article. Plasmid materials are available upon request for academic and non-profit research purposes.

## Results

### Design and efficient synthesis of challenging DNA sequences

To validate our established platform for synthesizing challenging ultralong sequences, we initially synthesized poly(T) sequences of varying lengths. Multiple synthesis reactions were designed to produce fragments ranging from 200 to 400 nt. PAGE analysis of the purified products revealed single bands of the expected sizes for all the samples (Fig. 2A), confirming the successful synthesis of the designed sequences.

STRs are widespread genomic elements that significantly impact gene regulation [26]. Among these, eSTRs represent a crucial subset that influences gene expression patterns. To further validate our synthesis platform, we randomly selected two eSTR loci with trinucleotide repeat units from previously identified eSTRs [27]. For each selected eSTR locus, we designed sequences incorporating 200 nt upstream and downstream of the genomic region, with varying copy numbers of eSTR repeat units determining the final sequence length. Specifically, we designed four oligonucleotide sequences of different lengths, containing 0, 17, 33, and 50 rpt, resulting in fragments of 400, 451, 499, and 550 nt, respectively. The designed sequences encompassed various challenging sequence types, including repetitive sequences, poly(T) sequences, and sequences with unbalanced base composition. Specifically, the *APAF1* interacting protein (*APIP*) gene–associated eSTR locus featured adenine–thymine (AT)-rich repetitive sequences and polyT sequences, while the *FRA10A* associated CGG repeat 1 (*FRA10AC1*) gene–associated eSTR locus contained guanine–cytosine (GC)-rich repetitive sequences. These final 400–550-nt oligonucleotides were synthesized using the method illustrated in Fig. 1. PAGE analysis verified that all the synthesized products exhibited their predicted molecular lengths (Fig. 2B), demonstrating the high accuracy of our synthesis platform. We aimed to investigate whether the length of intrinsic repetitive sequences in synthetic fragments could exceed 400 nt. To this end, we designed a 600-nt sequence comprising 270 consecutive cytosine–thymine (CT) repeats flanked by 30-nt homologous sequences at each terminus. The sequence was independently synthesized using four separate synthesis columns (*n* = 4 technical replicates) to verify platform stability. PAGE analysis confirmed successful synthesis of the target fragment with the expected molecular weight across all four replicates (Fig. 2C). These results demonstrate the reproducibility and consistency of our synthesis platform in generating ultralong challenging sequences.

### Selection and validation of synthetic DNA sequences

Despite the high coupling efficiency of 99.5% achieved in chemical DNA synthesis [28], the error accumulates progressively with increasing sequence length. For example, during the synthesis of a 300-nt sequence, the proportion of error-free fragments in the final product is reduced to ~22%. Consequently, the major downstream challenge lies in isolating these error-free sequences from the complex mixture of synthetic products. To address this limitation and ensure sequence accuracy, we implemented a purification strategy utilizing biotinylated primers complementary to the 5′ termini of target sequences. Following hybridization, we isolated full-length products using streptavidin-functionalized magnetic beads and employed enzymatic mismatch repair to eliminate error-containing sequences (Fig. 1C and D). The purified products were PCR-amplified and subcloned into appropriate vectors for subsequent analyses. Agarose gel electrophoresis revealed distinct, expected sized bands for all the synthetic sequences (Fig. 2D and E), while Sanger sequencing confirmed sequence accuracy across all the challenging motifs, regardless of length or complexity (Fig. S1A and B).

### Functional validation of synthetic STR sequences

To validate the biological function of our synthetic sequences, we employed two reporter gene systems to investigate their impact on gene expression. Firstly, we focused on the EGFP reporter system (Fig. 3A) to characterize the regulatory potential of our synthetic sequences. The backbone plasmid contained a minP driving the expression of EGFP, which served as the baseline control for comparative analysis. We cloned 400-nt and 451-nt synthetic sequences upstream of the EGFP gene, positioned immediately before the minP into the backbone plasmid. These two sequence groups differed only in the presence or absence of the eSTR locus. Following transfection into HEK293T cells, fluorescence microscopy revealed that cells transfected with plasmids containing our synthetic sequences derived from the *FRA10AC1* gene–associated eSTR loci exhibited significantly enhanced green fluorescent intensity compared to the control group. Notably, the sequence with eSTR sites demonstrated a more pronounced upregulation of EGFP expression than the sequence without eSTR loci (Fig. 3A). This enhanced EGFP expression suggests that our synthetic sequences possess robust regulatory capabilities in a cellular context. In contrast, the synthetic sequences derived from the *APIP* gene did not demonstrate a comparable upregulation effect (Fig. 3A). This discrepancy may be attributed to the AT-rich features of the sequences. Recent studies reveal that the dinucleotide repeat motif AT is depleted from enhancer sequences, suggesting that AT-rich sequences may play a nonessential or potentially inhibitory role in enhancer functionality [29].

Furthermore, we developed a dual-luciferase reporter system (Fig. 3B) to systematically investigate the relationship between the *FRA10AC1* gene–associated eSTR repeat numbers and downstream gene expression. We constructed plasmids incorporating 400-, 451-, and 499-nt sequences upstream of the luciferase reporter gene, positioned before the minP. These sequences corresponded to 0, 17, and 33 rpt, respectively. The backbone plasmid without any synthetic sequences inserted served as the control, and *Renilla* luciferase was used as an internal control. Quantitative analysis of luciferase activities in transfected HEK293T cells demonstrated a significant positive correlation between eSTR repeat numbers and luciferase expression (Fig. 3B). These results collectively indicate that our synthetic sequences not only maintain their regulatory function but also exhibit length-dependent modulation on downstream gene expression, with longer eSTRs conferring progressively stronger enhancement effects. The above functional analyses not only validated the accuracy of our synthetic sequences but also provided mechanistic insights into eSTR-mediated gene regulation.

### Precise design and validation of the synthetic biology element

After successfully validating the effectiveness of our DNA synthesis platform in generating STR sequences within the 400–600-nt length range, we further explored its potential applications in synthesizing important elements of synthetic biology. Specifically, we selected a biologically significant target sequence for synthesis: the 5×HRE [30, 31], which can be used to study cellular responses under hypoxic conditions. This element not only presents challenges in sequence complexity but also holds broad application prospects in synthetic biology.

The 5×HRE sequence consists of five repeated hypoxia-inducible factor binding sites (Fig. S2A). In synthetic biology, the 5×HRE can be utilized to develop expression systems for specific genes under hypoxic conditions, thereby enabling the regulation of gene function in specific environments. We synthesized a 5×HRE sequence (Fig. S2A) using the aforementioned DNA synthesis platform, which can be inserted upstream of specific gene sequences to induce expression in hypoxic environments. Sanger sequencing confirmed the precise synthesis (Fig. S2B). The successful synthesis of the 5×HRE sequence not only demonstrates the technical advantages of our DNA synthesis platform but also provides new tools and methodologies for research in synthetic biology.

## Discussion

The development of oligonucleotide synthesis technology holds significant importance for multiple fields, including synthetic biology and gene therapy. With the rapid advancement of life science technologies, DNA synthesis has become a critical technology driving continuous innovation. However, traditional DNA synthesis techniques are inherently limited in their capacity to synthesize sequences > 200 nt in length and complex sequences, consequently constraining research progress in synthetic biology and precision medicine. Faced with increasingly complex biological research demands, breaking through existing synthesis technology limitations has become an urgent necessity. To address this challenge, we established an ultralong-sequence synthesis platform based on nonporous solid silica microspheres. We functionalized solid, nonporous silica microspheres and utilized these preprocessed microspheres to construct a synthesis column that served as the reaction vessel for DNA synthesis. This platform innovatively combines capture technology through biotin primers and enzymatic error correction strategies, successfully overcoming the length limitations of traditional oligonucleotide synthesis. Utilizing this platform, we successfully synthesized complex sequences ≤ 600 nt in length. This breakthrough significantly expands the boundaries of oligonucleotide synthesis. Notably, the platform demonstrates exceptional capability in handling challenging complex sequences, including tandem repeat sequences, homopolymeric structures, and sequences with unbalanced base compositions.

We have also noted the emergence of other advanced strategies, such as CBP, which has demonstrated the potential for synthesizing ultralong genes, extending the length limit of phosphoramidite-based synthesis to 1728 nt [18]. Our present work builds on our prior success in ultralong oligonucleotide synthesis on silica substrates. We adapted an identical surface modification process, originally for planar silica microchips, to spherical solid silica microspheres. Although this rationale is shared with the CBP method (which uses borosilicate glass microspheres), our use of high-purity nonporous silica microspheres significantly boosts the subsequent synthesis performance. Critically, unlike CBP, which relies on specialized chemical tags and intricate gel purification, UCOS uses standard, commercially accessible biotin phosphoramidites. This approach, combined with simple downstream hybridization and magnetic bead–based capture, substantially reduces experimental costs and lowers the barrier to adoption across various research laboratories and industrial settings. While some methods focus on the absolute upper length limit of synthesis, the core focus of UCOS is to address the urgent demand for synthesizing ‘challenging genes’ (e.g. those with complex secondary structures, high repeat content, or uneven base composition) in synthetic genomics and genetic circuit construction. Furthermore, we demonstrate the biological functionality of the complex genetic elements synthesized using our platform, which is critical for their ultimate utility in microbial hosts. Collectively, this comprehensively optimized UCOS workflow effectively overcomes critical bottlenecks in complex gene construction, providing a valuable technical solution for advancing synthetic biology and accelerating the design–build–test cycle for complex systems.

The biotin primer–based capture technology and multistep enzymatic error correction method represent key innovations of our platform. Long oligonucleotide synthesis primarily requires consideration of two aspects. First is synthesis efficiency. Compared to fragments < 200 nt, the synthesis of longer oligonucleotides results in a significantly lower proportion of full-length fragments in the final product. A core technical challenge is purifying these low-quantity full-length fragments from the complex products. We designed a 20-nt reverse complementary primer targeting the gene's 5′ end, biotinylated at its 3′ terminus. When this biotin-labelled primer is annealed with synthesis products, it enables connecting successfully synthesized full-length fragments to streptavidin magnetic beads while removing non-full-length oligonucleotide fragments floating in the mixed system (Fig. 1C). Subsequently, low-cycle PCR amplification enriches these full-length fragments and releases the captured fragments from streptavidin magnetic beads. This step effectively improves the purity and accuracy of templates for subsequent enzymatic error correction (Fig. 1D), enhancing the efficiency of the error correction process and avoiding unexpected error fragments introduced by high template error rates.

Beyond full-length fragment enrichment and purification, long oligonucleotide synthesis must also consider synthesis accuracy. Previous literature reveals that common errors in oligonucleotide chemical synthesis include insertions, deletions, and substitutions, with corresponding error rates of 0.0045%, 0.1%, and 0.045% [32]. Consequently, during DNA chemical synthesis, the extension efficiency of each nucleotide incorporation is 99.8505% (100%—0.0045%—0.1%—0.045%). When synthesizing a 400-nt oligonucleotide, the theoretical final product accuracy is 54.97%. Actual accuracy may be even lower, with studies [33] indicating that the actual accuracy of 400-nt oligonucleotide synthesis is only 30%. Particularly when synthesizing G-base-rich sequences, the proportion of completely correct fragments significantly decreases. We analysed the accuracy of a 400-nt GC-rich sequence (64% GC content, *FRA10AC1*-400 nt) synthesized using our platform. Sanger sequencing results revealed a final product accuracy of 66.67%, far exceeding the theoretical accuracy (∼30%) for conventional synthesis. This result demonstrates that the multistep enzymatic error correction step in our fragment preparation process is highly effective and crucial, significantly improving the proportion of completely correct final products and avoiding redundant cloning screening steps resulting from high error rates.

**Figure 4:**
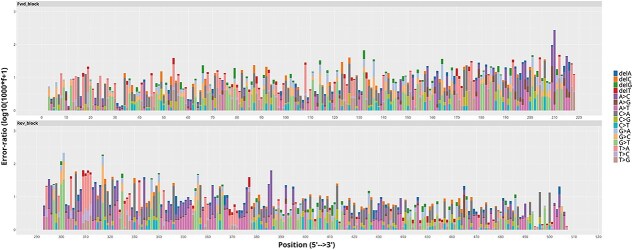
NGS-based accuracy analysis of the 510-nt GFP synthetic sequence. Bar chart showing the distribution of error types across different nucleotide positions. The x-axis represents the nucleotide positions along the 510-nt synthetic sequence, and the y-axis indicates the proportion of each error type (e.g. substitution, deletion) at the corresponding position. This panel illustrates the positional preference of errors and their relative abundance during synthesis.

To further confirm the reliability of the UCOS platform for long-sequence synthesis, we previously validated the accuracy of difficult-to-synthesize repetitive sequences using Sanger sequencing, achieving a 66.67% correct synthesis rate. To address the concern regarding comprehensive error rate assessment, we supplemented NGS-based verification for a 510-nt GFP-derived sequence and functional expression validation. Using 2 × 250 bp paired-end NGS sequencing, we analysed the error profile of the synthetic sequence (Fig. 4 and Tables S3 and S4), which revealed an average error rate as low as 1.2 errors per 1000 bases for the 510-nt fragment. Concurrently, functional validation by cloning the synthetic sequence into an expression vector revealed that ~63.71% of the colonies exhibited green fluorescence (Fig. S3), confirming the presence of correctly synthesized full-length sequences. These complementary validation approaches, including NGS-based error rate quantification and functional expression assessment, collectively demonstrate the robustness of the UCOS method, with consistent evidence supporting its ability to achieve reliable synthesis of long sequences. The high fidelity reflected by the ultralow error rate is consistent with the favourable functional validation results, further corroborating that UCOS maintains comparable accuracy to that observed for difficult-to-synthesize sequences reported previously.

While our enzyme-based mismatch repair effectively improves the synthesis accuracy, we also recognize potential limitations in the upstream PCR step—especially for highly repetitive sequences—which requires targeted optimization. We acknowledge that the initial PCR step in our workflow poses potential limitations when working with highly repetitive sequences. To mitigate these risks, we exclusively used our self-developed high-fidelity KOD DNA polymerase to minimize PCR-induced errors. The polymerase buffer contains a high magnesium ion concentration (optimized for AT-rich sequences), while for GC-rich sequences (e.g. *FRA10AC1*-associated eSTR loci) we supplemented the PCR system with 5% DMSO to eliminate secondary structures and ensure correct amplification. Despite these optimizations, extremely long repetitive regions (>200 base pairs of continuous repeats) may still present challenges—this limitation is noted to guide future integration of PCR-free amplification strategies.

**Figure 5:**
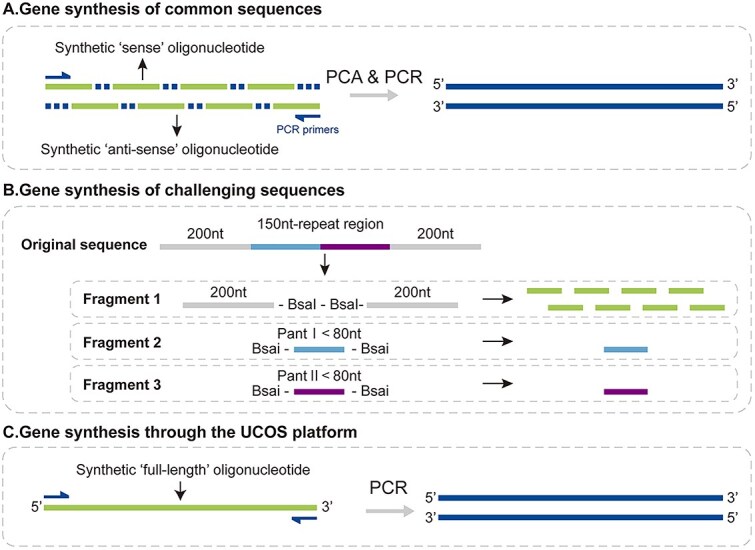
Comparison of conventional gene synthesis strategies and the long-fragment synthesis platform. (A) Schematic diagram of conventional gene synthesis for common sequences. The target sequence is divided into ~80-nt oligonucleotides with flanking sequences for bridging adjacent fragments. Synthesized sense and antisense sequences were assembled via polymerase cycling assembly (PCA). The complete synthetic fragment was then amplified by PCR. (B) Schematic diagram of gene synthesis for challenging sequences, exemplified by the 550-nt synthetic sequence in Fig. 2B. The full-length sequence is divided into two 200-nt flanking sequences (combined to form fragment 1) and one 150-nt repetitive region (divided into fragment 2 and fragment 3). These three fragments were synthesized independently and the correct fragments were ligated to obtain the full-length gene fragment. (C) Gene synthesis using the nonporous solid silica microsphere–based platform for long DNA fragments. Only a full-length sense sequence needs to be synthesized, which can then be PCR-amplified to obtain the complete synthetic fragment.

Direct synthesis of long oligonucleotide fragments can also conserve the reagents consumed in full-length gene synthesis. Traditional gene synthesis involves fragmenting the full-length gene into ~80-nt segments for synthesis and subsequent assembly (Fig. 5A), requiring near-duplicate synthesis of nearly half the gene sequence to achieve effective assembly. In contrast, our synthesis platform requires each gene sequence nucleotide to be synthesized only once, avoiding redundant synthesis at junction points and dramatically reducing reagent and solvent use (Fig. 5C). When synthesizing genes with repetitive sequences, conventional gene synthesis requires complex sequence fragmentation design and multiple synthesis steps (Fig. 5B). Even for sequences < 500 nt, two to three rounds of conventional gene synthesis are typically necessary. Our long-fragment synthesis platform, however, requires only a single synthesis cycle to obtain completely correct gene fragments or plasmids suitable for downstream applications (Fig. 5C).

Building on the reagent-saving advantage, our UCOS platform offers three key comprehensive advantages over conventional methods that address unmet needs in complex gene synthesis: (i) higher fidelity for complex sequences (66.67% accuracy for 400 nt GC-rich sequences, versus ~30% for conventional synthesis), (ii) reduced reagent consumption (no redundant synthesis of junction regions, cutting reagent use by ~50% for 500-nt sequences), and (iii) a shorter workflow time (50 hours for 300-nt challenging sequences, versus weeks for traditional methods). Regarding elongation efficacy (proportion of full-length products postsynthesis)—a key concern for long-oligo reliability—densitometric analysis of PAGE results (Fig. 2A–C) confirms that full-length fragments account for the majority of the initial synthesis products, with biotin enrichment further increasing the full-length purity. This high elongation efficacy not only supports reliable downstream gene assembly but also reduces the need for postsynthesis screening of non-full-length fragments, streamlining the overall workflow.

In addition to significantly reducing reagent consumption, our synthesis platform also effectively saves time costs. As illustrated in Fig. 1F, the detailed workflow and timeline of the UCOS synthesis platform are clear. For challenging sequences ~300 nt in length, although our synthesis time in column is somewhat longer compared to conventional gene synthesis, the total time required to obtain a correct sequence suitable for downstream functional validation is as short as 50 hours. In contrast, traditional methods often require two to three rounds of gene synthesis to address the challenges associated with DNA fragment assembly, resulting in an overall timeline typically measured in weeks.

To validate the time and cost advantages of our UCOS platform, we selected a challenging tandem repeat sequence featuring a 352-nt repetitive region from the genome (Fig. S4A). Full-length synthesis was performed using both UCOS and conventional services from vendor A. Within 8 days, UCOS delivered plasmids with perfectly synthesized sequences ready for downstream analysis (Fig. S4B), whereas vendor A failed to provide correct clones within their 28-day delivery window (Table 1). Additionally, vendor B declined to accept the synthesis order due to technical complexity, further underscoring the limitations of conventional services for challenging sequences. This practical comparison is further supported by systematic data on synthesis performance across methods (Table 1). Table 1 summarizes key parameters—including synthesis technology, time, cost, and quality—for UCOS and two commercial vendors (vendor A uses traditional gene synthesis; vendor B declines complex sequence orders). As shown, UCOS reduces synthesis time (8 days versus >28 days for vendor A) and cost (0.2 CNY per base versus 0.4 CNY per base), while achieving a 100% success rate for challenging sequences (versus 0% for vendor A). This combination of practical validation and systematic parameter comparison confirms that UCOS outperforms conventional commercial services in addressing the core pain points of complex DNA synthesis.

**Table 1:** Comparison between the UCOS platform and the currently commercially available services

Vendors	Synthesis technology	Time	Cost	Quality
This study	UCOS	8 days	0.2 CNY per base	Success
Vendor A	Traditional gene synthesis of challenging genes	>28 days	0.4 CNY per base	Failure
Vendor B	Traditional gene synthesis of challenging genes	N/A	N/A	Refusal to accept orders

While our current results validate the core performance of the UCOS platform, we acknowledge areas for further refinement in experimental design to enhance robustness. We acknowledge that some experiments (e.g. synthesis of the CT-rich sequence) were conducted with four technical replicates (consistent with Fig. 2C showing four independent columns for 600-nt sequences), with consistent results supporting reliability. For functional validation, testing in HEK293T cells was prioritized as a well-established model for reporter gene assays; future studies will include additional cell types (e.g. HeLa) and time-course experiments (24–72 hours posttransfection) to further confirm regulatory effects.

In summary, we have established a robust and practical workflow that encompasses long-fragment synthesis, accurate sequence selection, plasmid construction, and subsequent functional validation. In this study, we successfully employed this newly established synthesis platform to generate STRs with repeat units of 1–6 nt (Fig. S5A). Nevertheless, this achievement does not signify the upper limit of the platform's capabilities. A variety of naturally occurring yet highly complex sequences—many of which are difficult to synthesize via existing technologies—can also be synthesized using our platform. Notably, there are more tandem repeats in the genome with repeat units > 6 nt (Fig. S5B), exhibiting high copy numbers. Our platform holds promise for advancing in-depth investigations of these loci at their native copy numbers. Moreover, the *de novo* design of some regulatory elements, such as 3′ UTRs and circular RNAs, can require the synthesis of fully randomized sequences ≥ 250 nt (Fig. S5C) for screening and validation [34]. Many protein-coding genes also harbour repetitive motifs that are important for synthetic biology. For instance, synthesizing the pentapeptide sequence VPGVG—derived from elastin—to produce elastin-like polypeptides (ELPs) is critical. ELPs are high-value bioproducts whose synthesis relies on constructing these long, challenging tandem repeats (Fig. S5D) [35], which alter their solubility with temperature changes and thus have broad potential applications in biomedicine and drug delivery. Similarly, synthesizing the repeat motifs of transcription activator–like effector (TALE) (Fig. S5E) is especially challenging, as TALEs contain multiple repeats of a 34–amino acid sequence [36], with each repeat recognizing a specific DNA base through repeat variable diresidues (RVDs). Spider silk protein genes offer yet another example of naturally intricate protein-coding sequences. Their cDNA can span up to 2.4 kb and includes numerous repeats rich in glycine and alanine residues (Fig. S5F) [37], with the longest repeat unit reaching 34 amino acids (equivalent to 102 nucleotides). Taken together, the demonstrated capability of UCOS to reliably synthesize ultralong and complex DNA sequences is a significant step. UCOS enables the streamlined creation of these complex genetic blueprints, facilitating protein engineering and biomanufacturing efforts.

It should be noted that the experimental validation of this study is limited to the successful synthesis of oligonucleotides containing STRs. The sequences depicted in Fig. S5, which are featured with long lengths and high repeat densities, remain challenging targets for the current UCOS platform—these characteristics lead to multiple technical hurdles, including reduced synthesis coupling efficiency, inefficient postsynthesis amplification due to secondary structure formation, and increased difficulty in accurate sequencing. Therefore, the primary future direction of the UCOS platform will focus on optimizing the workflow to overcome these challenges, with the core goal of achieving efficient and high-fidelity synthesis of the refractory sequences described in Fig. S5. Further refinements will enable rapid assembly of these challenging constructs, opening new avenues for fundamental biomedical research and practical applications, especially in synthetic genomics, complex circuit assembly, and the high-throughput development of genetic devices.

## Supplementary Material

Supplementary_Material_ysag005

## Data Availability

The data that support the findings of this study are available from the corresponding author upon reasonable request.
