# Supplementary material for: Chemically synthesized high-fidelity oligos ≤ 600 nt as building blocks to accelerate complex gene construction in synthetic biology
Source: Synth Biol (Oxf). 2026 May 23;11(1):ysag005. doi: 10.1093/synbio/ysag005 (PMC13218287; doi:10.1093/synbio/ysag005)
Supplement: Supplementary_Material_ysag005 [file supplementary_material_ysag005.zip › Supplementary_Material_ysag005.pdf]

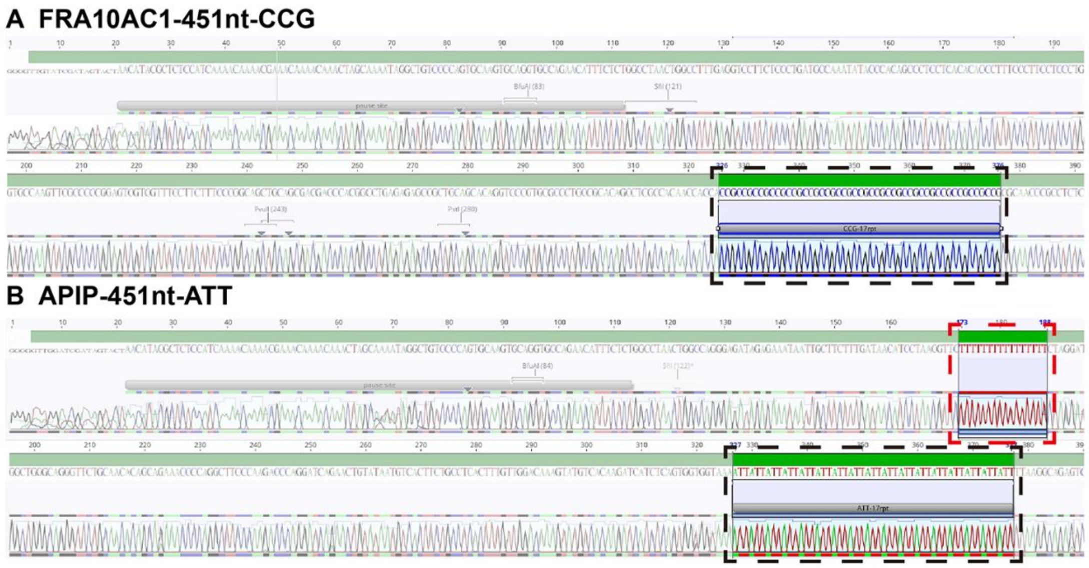

Figure S1. Validation of synthetic eSTR-containing DNA sequences.

(A-B). Sanger sequencing validation of synthetic FRA10AC1-related 451nt DNA sequences (A) and APIP-related 451nt DNA sequences (B). The black boxes indicate the repeat sequences (CCG or ATT) in each group, while the red box highlights the poly(T) sequences.

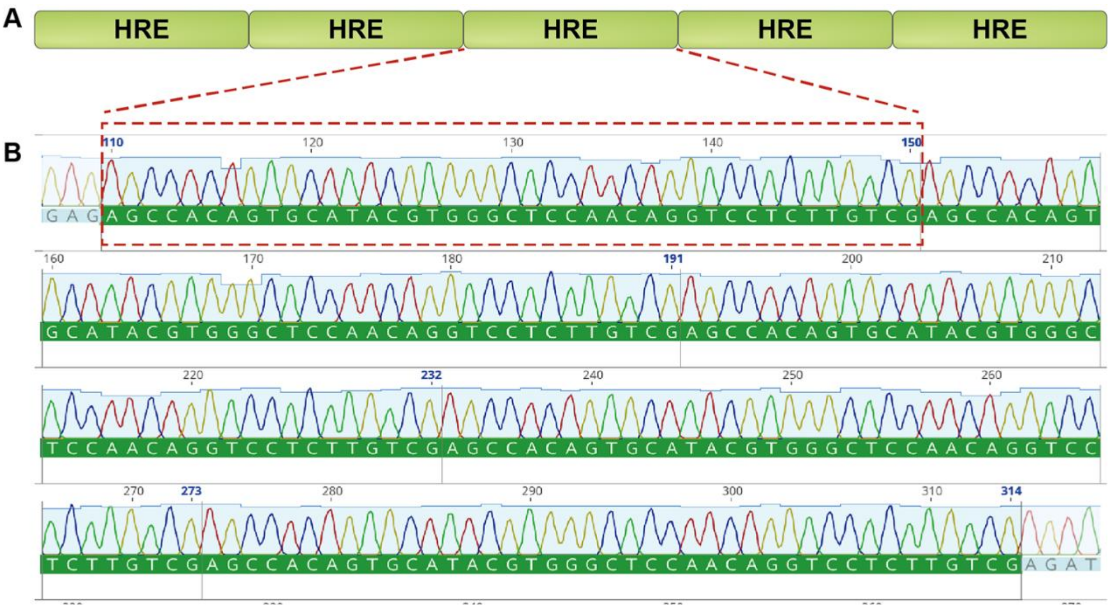

Figure S2. Validation of the synthesized 5xHRE sequence.

(A). Schematic diagram of the 5xHRE sequence

(B). Sanger sequencing validation of the synthetic 5xHRE sequence. Bases 110–150, 151–191, 192–232, 233–273, and 274–314 each represent one of the five distinct HRE sequence frameworks.

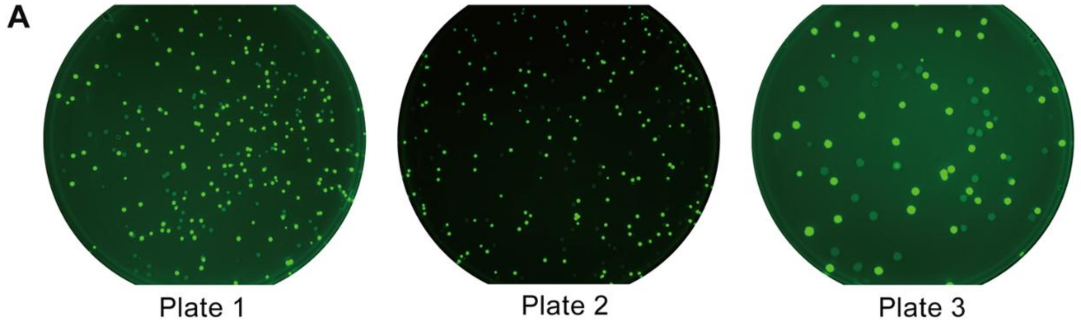

B

	Plate 1	Plate 2	Plate 3	Total
Positive colony	162	171	48	381
Negative colony	89	97	31	217
Tatol colony	251	268	79	598
Percent	64.54%	63.81%	60.76%	63.71%

Figure S3. Validation of synthetic 510nt GFP-derived sequences.

(A). Fluorescence imaging of bacterial colonies on three independent culture plates. Colonies expressing green fluorescent protein (GFP) indicate successful synthesis and functional integration of the target sequence, while non-fluorescent colonies represent either incorrect synthesis or failed expression.

(B). Statistical table summarizing colony counting results from the three plates. The table includes the number of positive clones (fluorescent), negative clones (non-fluorescent), and the corresponding positive clone ratio for each plate. Cumulative analysis shows that the proportion of functionally correct fluorescent colonies (with accurate sequences) reaches 63.71%, consistent with the NGS-based accuracy verification results and further confirming the reliability of the UCOS synthetic platform.

A

1	TGACTCTACC	CTTGCATAGG	TAGTTTTCTT	TCCTTCCTTC	CTTCCTTCCT	TCCTTCCTTC
61	CTTCCTTCCT	TCCTTCCTTC	CCTTCCTTTT	TTTCTTTTCT	CTTCTCTTCC	CTTCCCCTCC
121	CTCCCTCTCT	TTCTCCCTTT	CTCCCTTCCC	TTTCCTTTCA	GTTCCGTTCC	TTTCCTCTTC
181	ATTTTTTCTT	CCTTCCTTCC	TTCCTTCTTT	CTCCCTTCCC	CTCCCTTCCC	TTCCCCTTCT
241	TTCTCCCTTC	CCCTCCCTTC	CCTTCCTTTC	CCCTTCCCCT	CCCTTCCCTT	CCCTTCCCCT
301	TCTCCTTCCT	TCCTTCCTTC	CTTACTTCTC	TCTTTCTTTT	TTGGTTGAGA	CA

B

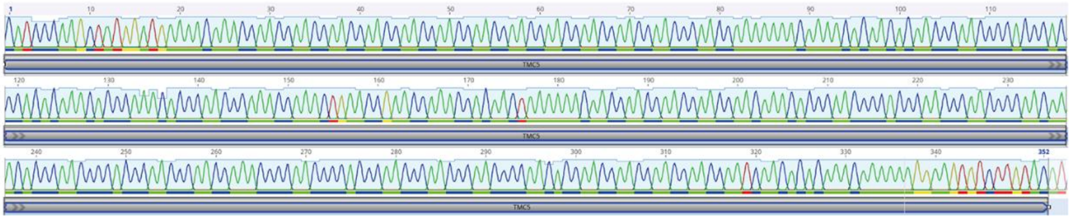

Figure S4. Validation of synthetic 352nt DNA sequences.

(A). Detail information of the 352nt tandem repeat sequence.

(B). Sanger sequencing validation of the synthetic 352nt DNA sequence.

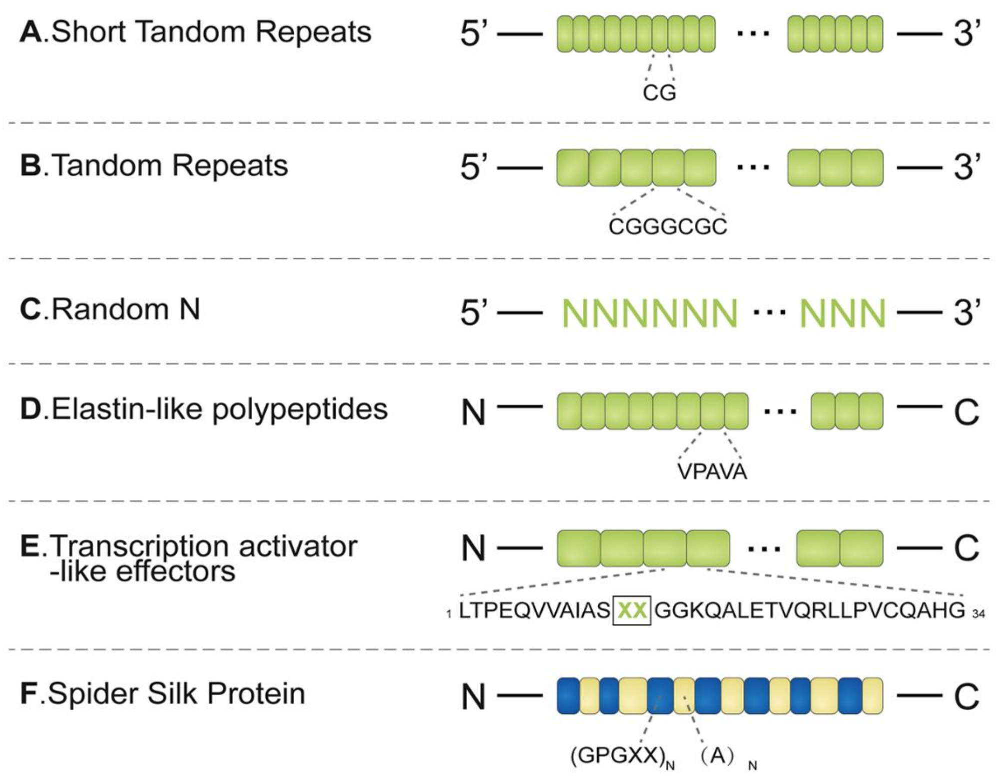

Figure S5. Schematic illustration of potential complex and challenging sequences that can be synthesized using our platform.

(A). Short tandem repeat sequences. The repeat unit is in the 1–6 nt range and typically exhibits an imbalanced base composition.

(B). Tandem repeat sequences. The repeat unit is longer than 6 nt and generally also exhibits an imbalanced base composition.

(C). Long random sequences. De novo design of regulatory elements such as circular RNAs or 3′UTRs often requires the synthesis of fully random sequences of 250 nt or more.

(D). Elastin-like polypeptides. The temperature sensitivity of these proteins depends primarily on longer sequences formed by tandem repeats of a pentapeptide motif.

(E). Transcription activator-like effectors. The core region of these proteins comprises

tandem repeats of a 34-amino-acid sequence that is nearly identical. The specific nucleotide (A, T, C, or G) recognized by TALE is determined by the 12th and 13th residues, known as repeat variable di-residues (RVDs).

(F). Spider silk proteins. These natural protein-coding genes are enriched in glycine and alanine residues and contain numerous tandem repeats.

Table S1. Detailed information for synthetic sequences.

Gene ID	Sequences
APIP-<br>400nt	AGGGAGATAGAGAAATAATTGCTTCTTTGATAACATCCTAACGTTCTT<br>TTTTTTTTTTTTTTCTAGGATGGCTGGGCAGGGTTCTGCAACACAGCAG<br>AAACCCCAGGCTTCCCAAGACCCAGGATCAGAACTGTATAATGTCACT<br>TCTGCCTCACTTTGTTGGACAAAGTATGTCACAAGATCATCTCAGTGGT<br>GGTAAATTAAGGCAGAGTCTCACTTTGTCACCCAGGCTGGAGTGCAGT<br>GGTGTGATCTCGGCTGCTCACTACAACCTCCGCCTCCCATGTTAAGCG<br>ACTCTCATGCCTCAACCTTACCCATGTTAAGCGACTCTCATGCCTCAAC<br>CTTACCAAGTAGCTGGGATTACAGGTGCATGCCACCACAGCTGGCTAA<br>ATTTTGTATTTTT
APIP-<br>451nt	AGGGAGATAGAGAAATAATTGCTTCTTTGATAACATCCTAACGTTCTT<br>TTTTTTTTTTTTTTCTAGGATGGCTGGGCAGGGTTCTGCAACACAGCAG<br>AAACCCCAGGCTTCCCAAGACCCAGGATCAGAACTGTATAATGTCACT<br>TCTGCCTCACTTTGTTGGACAAAGTATGTCACAAGATCATCTCAGTGGT<br>GGTAAAAttattattattattattattattattattattattattattattattattattTTAAGGCAGAGTCTCA<br>CTTTGTCACCCAGGCTGGAGTGCAGTGGTGTGATCTCGGCTGCTCACT<br>ACAACCTCCGCCTCCCATGTTAAGCGACTCTCATGCCTCAACCTTACCC<br>ATGTTAAGCGACTCTCATGCCTCAACCTTACCAAGTAGCTGGGATTAC<br>AGGTGCATGCCACCACAGCTGGCTAAATTTTGTATTTTT
APIP-<br>499nt	AGGGAGATAGAGAAATAATTGCTTCTTTGATAACATCCTAACGTTCTT<br>TTTTTTTTTTTTTTCTAGGATGGCTGGGCAGGGTTCTGCAACACAGCAG<br>AAACCCCAGGCTTCCCAAGACCCAGGATCAGAACTGTATAATGTCACT<br>TCTGCCTCACTTTGTTGGACAAAGTATGTCACAAGATCATCTCAGTGGT<br>GGTAAAAttattattattattattattattattattattattattattattattattattattattattattattattattattattatta<br>ttattattattattTTAAGGCAGAGTCTCACTTTGTCACCCAGGCTGGAGTGCAG<br>TGGTGTGATCTCGGCTGCTCACTACAACCTCCGCCTCCCATGTTAAGCG<br>ACTCTCATGCCTCAACCTTACCCATGTTAAGCGACTCTCATGCCTCAAC<br>CTTACCAAGTAGCTGGGATTACAGGTGCATGCCACCACAGCTGGCTAA<br>ATTTTGTATTTTT
APIP-<br>550nt	AGGGAGATAGAGAAATAATTGCTTCTTTGATAACATCCTAACGTTCTT<br>TTTTTTTTTTTTTTCTAGGATGGCTGGGCAGGGTTCTGCAACACAGCAG<br>AAACCCCAGGCTTCCCAAGACCCAGGATCAGAACTGTATAATGTCACT<br>TCTGCCTCACTTTGTTGGACAAAGTATGTCACAAGATCATCTCAGTGGT<br>GGTAAAAttattattattattattattattattattattattattattattattattattattattattattattattattattattatta<br>ttattattattattattattattattattattattattattattattattattattattattattTTAAGGCAGAGTCTCA<br>CTTTGTCACCCAGGCTGGAGTGCAGTGGTGTGATCTCGGCTGCTCACT<br>ACAACCTCCGCCTCCCATGTTAAGCGACTCTCATGCCTCAACCTTACCC<br>ATGTTAAGCGACTCTCATGCCTCAACCTTACCAAGTAGCTGGGATTAC<br>AGGTGCATGCCACCACAGCTGGCTAAATTTTGTATTTTT
FRA10-<br>400nt	TTTGAGGTCCTTCTCCCTGATGCCAAATATACCCACAGCCCTCCTCACA<br>CACCCTTTCCCTTCCTCCCTGTGTGCCAAGTTCGCCCCCGGAGTCGTCG<br>TTTCCTTCTTTCCCGGCAGCTGCAGCGACGACCCACGGCCTGAGAGAG

	CCGCTGCAGCACAGGTCCCGTGCGCCCTGCCGCACAGCCTCGCCACAA<br>CCACCACGCAACCCGCCTCTCCCTACGGGTCCCGACTGGGCACCACTT<br>CCGGTCCGACACGGCCACGTGTTACATCTAAATGGCACCGTCCCCCGA<br>GTGCGCCGACCTTGTGCTACAGCCAGAACAGCTCCGGGAAACAAGGG<br>CAGCGGGAGAAACGGCCCGGAAAGGGACGAAGAGGCTTCCAGCTTTC<br>GCCAAGTCGCGGCTCC
FRA10-451nt	TTTGAGGTCCTTCTCCCTGATGCCAAATATACCCACAGCCCTCCTCACA<br>CACCCTTTCCCTTCCTCCCTGTGTGCCAAGTTCGCCCCCGGAGTCGTCG<br>TTTCCTTCTTTCCCGGCAGCTGCAGCGACGACCCACGGCCTGAGAGAG<br>CCGCTGCAGCACAGGTCCCGTGCGCCCTGCCGCACAGCCTCGCCACAA<br>CCACCACcgccgccgccgccgccgccgccgccgccgccgccgccgccgccgccgccgCGCAACC<br>CGCCTCTCCCTACGGGTCCCGACTGGGCACCACTTCCGGTCCGACACG<br>GCCACGTGTTACATCTAAATGGCACCGTCCCCCGAGTGCGCCGACCTT<br>GTGCTACAGCCAGAACAGCTCCGGGAAACAAGGGCAGCGGGAGAAAC<br>GGCCCGGAAAGGGACGAAGAGGCTTCCAGCTTTCGCCAAGTCGCGGC<br>TCC
FRA10-499nt	TTTGAGGTCCTTCTCCCTGATGCCAAATATACCCACAGCCCTCCTCACA<br>CACCCTTTCCCTTCCTCCCTGTGTGCCAAGTTCGCCCCCGGAGTCGTCG<br>TTTCCTTCTTTCCCGGCAGCTGCAGCGACGACCCACGGCCTGAGAGAG<br>CCGCTGCAGCACAGGTCCCGTGCGCCCTGCCGCACAGCCTCGCCACAA<br>CCACCACcgccgccgccgccgccgccgccgccgccgccgccgccgccgccgccgccgccgccgccgcc<br>gccgccgccgccgccgccgccgccgccgccgccgccgCGCAACCCGCCTCTCCCTACGGG<br>TCCCGACTGGGCACCACTTCCGGTCCGACACGGCCACGTGTTACATCT<br>AAATGGCACCGTCCCCCGAGTGCGCCGACCTTGTGCTACAGCCAGAAC<br>AGCTCCGGGAAACAAGGGCAGCGGGAGAAACGGCCCGGAAAGGGAC<br>GAAGAGGCTTCCAGCTTTCGCCAAGTCGCGGCTCC
FRA10-550nt	TTTGAGGTCCTTCTCCCTGATGCCAAATATACCCACAGCCCTCCTCACA<br>CACCCTTTCCCTTCCTCCCTGTGTGCCAAGTTCGCCCCCGGAGTCGTCG<br>TTTCCTTCTTTCCCGGCAGCTGCAGCGACGACCCACGGCCTGAGAGAG<br>CCGCTGCAGCACAGGTCCCGTGCGCCCTGCCGCACAGCCTCGCCACAA<br>CCACCACcgccgccgccgccgccgccgccgccgccgccgccgccgccgccgccgccgccgccgccgcc<br>gccgccgccgccgccgccgccgccgccgccgccgccgccgccgccgccgccgccgccgccgccgccgccgc<br>cgccgccgccgccgccgCGCAACCCGCCTCTCCCTACGGGTCCCGACTGGGCAC<br>CACTTCCGGTCCGACACGGCCACGTGTTACATCTAAATGGCACCGTCC<br>CCCGAGTGCGCCGACCTTGTGCTACAGCCAGAACAGCTCCGGGAAACA<br>AGGGCAGCGGGAGAAACGGCCCGGAAAGGGACGAAGAGGCTTCCAG<br>CTTTCGCCAAGTCGCGGCTCC
600nt-CTrepeat	CGACGTTGTAAAACGACGGCCAGTGAATTCctctctctctctctctctctctctctctctctct<br>ctctctctctctctctctctctctctctctctctctctctctctctctctctctctctctctctctctctctctctctctctctctctctctctc<br>tctctctctctctctctctctctctctctctctctctctctctctctctctctctctctctctctctctctctctctctctctctctctctctct<br>ctctctctctctctctctctctctctctctctctctctctctctctctctctctctctctctctctctctctctctctctctctctctctctctc<br>tctctctctctctctctctctctctctctctctctctctctctctctctctctctctctctctctctctctctctctctctctctctctctctct<br>ctctctctctctctctctctctctctctctctctctctctctctctctctctctctctctctctctctctctctctctctctctctctctctctc

	tctctctctctctctctctctctctctctctctctctctctctctctctctAAGCTTGGCGTAATCATGGTCATAGCTGTT
510nt-GFP	CTGACGCTGAAGTTCATCTGTACTACTGGTAAACTGCCGGTACCTTGGCCGACTCTGGTAACGACGCTGACTTATGGTGTTCAGTGCTTTGCTCGTTATCCGGACCATATGAAGCAGCATGACTTCTTCAAGTCCGCCATGCCGGAAGGCTATGTGCAGGAACGCACGATTTCCTTTAAGGATGACGGCACGTACAAAACGCGTGCGGAAGTGAAATTTGAAGGCGATACCCTGGTAAACCGCATTGAGCTGAAAGGCATTGACTTTAAAGAAGACGGCAATATCCTGGGCCATAAGCTGGAATACAATTTTAACAGCCACAATGTTTACATCACCGCCGATAAACAAAAAAATGGCATTAAAGCGAATTTTAAAATTCGCCACAACGTGGAGGATGGCAGCGTGCAGCTGGCTGATCACTACCAGCAAAACACTCCAATCGGTGATGGTCCtGTTCTGCTGCCAGACAATCACTATCTGAGCACGCAAAGCGTTCTGTCTAAAGATCCG

Table S2. Detailed information for primers.

Application	Primer ID	Sequences
primers for oligo capture	APIP-Biotin	AATTATTTCTCTATCTCCCT
	FRA10-Biotin	TCAGGGAGAAGGACCTCAAA
	600nt-Biotin	GCCGTCGTTTTACAACGTCG
	510nt-Biotin	CAGATGAACTTCAGCGTCAG
primers for PCR after capture	APIP-F	AGGGAGATAGAGAAATAATTGCTTCT
	APIP-R	AAAAATACAAAATTTAGCCAGCTGTG
	FRA10-F	TTTGAGGTCCTTCTCCCTGATGCCAA
	FRA10-R	GGAGCCGCGACTTGGCGAAA
primers for PCR after correction	APIP-Gibson-F	acatttctctggcctaactggccAGGGAGATAGAGAAATAATTGCTTCT
	APIP-Gibson-R	gtctaagcttggccgccgaggccAAAAATACAAAATTTAGCCAGCTGTG
	FRA10-Gibson-F	acatttctctggcctaactggccTTTGAGGTCCTTCTCCCTGATGCCAA
	FRA10-Gibson-R	gtctaagcttggccgccgaggccGGAGCCGCGACTTGGCGAAA
primers for PCR of 600nt-CTrepeat	1/2 PCR-PUC57-FP-F	GTTGTAAAACGACGGCCAGT
	1/2 PCR-PUC57-FP-R	AACAGCTATGACCATGATTACGC
primers for PCR of 510nt-GFP	510nt-F	CTGACGCTGAAGTTCATCTGTACTAC
	510nt-R	CGGATCTTTAGACAGAACGCTT

Table S4. Match ratios of the 510nt GFP sequence.

/	**Total_pair**	**R1_match**	**R2_match**
Count	83300	53946	50736
Percent	/	64.76%	60.91%
